# Identifying and Responding to Delirium in Acute Stroke: Clinical Team Members’ Understandings

**DOI:** 10.1177/1049732320959295

**Published:** 2020-09-24

**Authors:** Gail Carin-Levy, Kath Nicol, Frederike van Wijck, Gillian Mead, Chris McVittie

**Affiliations:** 1 Queen Margaret University Edinburgh, Musselburgh, United Kingdom; 2 Glasgow Caledonian University, Glasgow, United Kingdom; 3 The University of Edinburgh, Edinburgh, United Kingdom

**Keywords:** delirium, acute stroke, interprofessional care, focus groups, constructivist grounded theory, qualitative, Scotland

## Abstract

Delirium is associated with increased mortality, morbidity, and length of hospital stay. In the acute stroke setting, delirium identification is challenging due to the complexity of cognitive screening in this patient group. The aim of this study was to explore how members of interprofessional stroke-unit teams identified and responded to a potential delirium in a patient. Online focus groups and interviews utilizing case vignettes were conducted with 15 participants: nurses, occupational therapists, speech and language therapists, and physiotherapists working in acute stroke services. Participants’ understandings of delirium varied, most participants did not identify the symptoms of a possible hypoactive delirium, and nearly all participants discussed delirium symptoms in tentative terms. Aspects of interprofessional working were discussed through the expression of distinct roles around delirium identification. Although participants demonstrated an ethos of person-focused care, there are ongoing challenges involved in early identification and management of delirium in stroke survivors.

## Introduction

Delirium is a complex neuropsychiatric condition characterized by acute onset and fluctuating disturbance of attention, cognition, perception, motor behavior, and sleep–wake patterns (American Psychiatric Association, 2013). Delirium can present as hyperactive, hypoactive, or an unpredictable fluctuation between the two. The hyperactive form is characterized by agitation, distractibility, and overt psychotic symptoms, whereas the hypoactive form is characterized by sedation, withdrawal, and is often missed as a diagnosis due to this manifestation (Young & Inouye, 2007). The etiology of delirium is regarded as nonspecific, but often there are multiple, underlying causes for the condition (Inouye et al., 2014). Stroke survivors often possess a number of the risk factors associated with developing delirium (Oldenbeuving et al., 2014); indeed, it is found to affect 26% to 28% of patients in the acute stroke setting (Carin-Levy et al., 2012; Shi et al., 2012). Stroke survivors who develop delirium are affected by significantly poorer outcomes compared with patients who do not: increased 12-month mortality, poorer functional outcomes, and an increased risk of developing dementia (Carin-Levy et al., 2012; Shi et al., 2012).

Early identification is considered key in the effective management of delirium (Holly et al., 2013), yet delirium recognition is a challenge irrespective of the country of practice or hospital setting (Bhat & Rockwood, 2016; Rice et al., 2011; Ryan et al., 2013). The barriers to effective and timely delirium identification have been studied internationally, and in the practice of doctors and nurses, a lack of awareness of the seriousness of the condition and its prevalence as well as a lack of confidence were found to be responsible for low identification rates (Baker et al., 2015; Davis & MacLullich, 2009; Ettema et al., 2014; Flagg et al., 2010; Schuurmans et al., 2001). The challenge of delirium identification is heightened within acute stroke due to the difficulties associated with cognitive screening in patients who are acutely unwell, experiencing aphasia or other cognitive difficulties arising from the stroke itself (Infante et al., 2017; Lees et al., 2013), yet, to date, no empirical evaluation of these challenges appears to have been published.

Effective management of delirium in any hospital setting relies on interprofessional education programs to facilitate the implementation of multicomponent, interprofessional approaches to delirium management (Abraha et al., 2015; Hshieh et al., 2015; Siddiqi et al., 2016). Despite the clear need for interprofessional practice, literature published on the topic has thus far been concerned particularly with the role of doctors and nurses, with little attention paid to the role of other professionals who are part of interprofessional teams. This is demonstrated clearly in Sockalingam et al.’s (2014) systematic review of interprofessional education for delirium care, most of the studies included in this review were of doctors and nurses, and only a handful of the studies had a therapist included in the professional mix. It is recognized that nurses are best placed to assume a role in delirium identification due to their consistent contact with patients over a 24-hour cycle (Hall et al., 2012). However, this role could extend to clinicians from other disciplines such as physiotherapists, occupational therapists, and speech and language therapists, particularly in a stroke setting, where close and effective interprofessional team-working is widely recognized (Clarke, 2010). We sought therefore to fill this specific gap in the literature on interprofessional delirium identification by exploring whether and how clinicians within a team might recognize delirium. The aim of this study was to examine how professionals working within interprofessional teams would respond to cases of potential delirium in stroke survivors.

## Method

### Data Collection

The study was conducted in Scotland, UK. This site was especially relevant for the current study as the identification and early management of delirium has, over recent years, been a health care priority for the Scottish Government as part of its efforts to improve the care for older people, reflected in its “Think Delirium” toolkit (Healthcare Improvement Scotland, 2014).

Staff from a variety of professional disciplines working in stroke units across Scotland were targeted for recruitment. Contact was made by emailing clinical and special interest groups and professional associations, as well as generating publicity via social media networks. To avoid priming potential participants of the aim of the study, recruitment materials did not refer to delirium but instead to “psychological difficulties.” Medical professionals, nurses, occupational therapists, physiotherapists, and speech and language therapists who were employed by National Health Service (NHS) Scotland in a clinical capacity in an acute stroke environment were invited to contact the first author for further details. Fifteen respondents who met the above criteria agreed to participate in the study. Participants worked in stroke units of sizes that varied between six and 40 beds, and had experience ranging in length from less than 1 year to 20 years. The final sample comprised three nurses, five occupational therapists, two physiotherapists, and five speech and language therapists. Two participants, namely, one nurse and one physiotherapist, had shortly prior to the study received specific training in delirium; other participants had not received such training. Participants provided informed written consent to take part in the study.

The data were generated using online, asynchronous focus groups. Online focus groups can generate rich data that are of similar quality to those obtained from face-to-face groups (Woodyatt et al., 2016) and are particularly effective in allowing participation by individuals who are geographically dispersed and who cannot readily meet in one place (Matthews et al., 2018). In the present case, online groups were used to facilitate the participation of professionals who were in employment in diverse locations. Discussions were hosted on the virtual learning environment Blackboard CourseSites®, which allowed each participant to create a username and to post anonymously to the discussions. To guide group discussions, we used vignettes, a method that has been found to be effective in facilitating group discussions of potentially difficult topics (Owens et al., 2018). Here, the vignettes used were modeled upon Fick et al.’s (2013) work which used standardized case vignettes to explore delirium knowledge and recognition in nurses. These vignettes were adapted for use in a stroke environment. Once approved by the team, these were sent to a consultant liaison psychiatrist and a consultant stroke physician for final approval. The final versions of the vignettes were based on actual clinical manifestations of patients with a diagnosis of delirium following a stroke. The first scenario depicted a female patient exhibiting symptoms associated with hypoactive delirium, and the second scenario depicted a male patient exhibiting symptoms associated with hyperactive delirium. The baseline information for both case scenarios was the same. The vignettes and the schedule of questions were approved by a consultant stroke physician and a consultant in liaison psychiatry. (The vignettes used are included in the supplemental file.)

The two focus groups finally comprised seven and five participants. Three participants, who found engagement with the host platform difficult, withdrew from the groups prior to commencement. Group discussions were moderated over a period of 2 months. Participants were presented at successive intervals with further details from each vignette with the first scenario (hypoactive delirium) presented and discussed in full before moving onto the second scenario (hyperactive delirium). Participants were asked to respond to open-ended questions relating to the information made available to date, a method of vignette utilization reported by Jenkins et al. (2010). Participants could view each other’s contributions and could post as much and as often as they liked to the discussions. Once discussions were concluded, all participants were provided with information on delirium and were invited to add any comments. The three participants who had withdrawn from the group discussions subsequently participated in email interviews, conducted along similar lines to the group discussions in terms of utilizing the case vignettes as conduit to interview discussions. As these three participants did not engage with the online focus group platform, they were unable to see other participants’ comments. Although this inevitably resulted in a different experience, these participants engaged with the vignettes to an extent similar to the focus group discussions and their contributions provided useful data from key members of clinical teams.

### Data Analysis

Analysis drew upon the principles of constructivist grounded theory (Charmaz, 2014) to explore clinicians’ understandings of delirium in stroke survivors. As an inductive approach, grounded theory emphasizes the generation of theory from close inspection of qualitative data and is particularly appropriate for inquiry into topics where little previous work has been conducted and theoretical development is required. Constructivist grounded theory proceeds from a relativist ontological standpoint, emphasizing the ways in which individuals themselves construct the reality of the phenomena under consideration. The epistemological approach thus is an interpretative one. It requires the engagement of the researcher with the fine-grained detail of the data and the research process to derive understandings of the phenomena under investigation that are grounded in the participants’ contextual experiences of what is being described. This foregrounds the inductive quality of the research and allows for the emergence of understandings that are demonstrably relevant for the participants in their descriptions of everyday experiences of the phenomena (Charmaz, 2014). Here, the transcripts of the group discussions and email interviews were coded utilizing *N-Vivo* (version 10.0) to identity initial indicators of meaning for the participants. Line-by-line coding was carried out and links between the descriptive codes were established and codes were synthesized into emergent categories. Memo writing to maintain a record of potential links between codes, reflexivity on the developing synthesis, and constant comparison between cases were used to refine and extend emerging categories (Charmaz, 2014).

In line with recognized principles of grounded theory, initial analysis of data from the first focus group informed the collection of data from the second focus group, and subsequent email interviews were informed by initial analysis of all data collected to that point. Drawing on this form of theoretical sampling, a key element of grounded theory (Strauss, 1988) allowed for categories to be extended and deepened as the study progressed. Analysis was conducted on a recursive basis, with emerging categories being adapted and refined in light of potentially negative cases and compared across all codes within the data set. One such example is found in the first category discussed below. Initial analysis indicated that, in discussing the case depicted in the vignette, most participants did not refer to delirium. Subsequent codes, however, showed that the two participants who had received delirium training did use this term. These references thus could be regarded as negative cases that challenged the emerging analysis. Further analysis showed that although these two participants introduced the term delirium, they did not offer it as an explanation for the symptoms that were described. Analysis of these two cases, along with those of other participants, therefore led to a revised category of “Uncertainty around symptom recognition” that accounted for all cases in the data set. This analytic process led to the emergence of four categories that demonstrated best analytic fit for the data. Final analysis was agreed by the full research team.

In the extracts produced below, typographical errors in the data have been corrected and abbreviations expanded to improve readability of the data. Otherwise, the extracts comprise the participants’ own words and demonstrate fidelity to their descriptions of the topics that were being discussed. The inclusion of these data alongside the analysis being provided allows readers to evaluate for themselves the analytic claims being made and the value of the analysis in addressing the aim of the study. The present article thus demonstrates “methodological integrity,” the criterion proposed by Levitt et al. (2017) for evaluating the quality of a qualitative research study such as this one.

### Ethical Considerations

Ethical approval was gained from a University Ethics Committee. The study protocol was sent to the Research and Development department of the local NHS board and exempt from full ethical review.

## Results

The four categories that emerged from the analysis described above were as follows: (a) uncertainly around symptom recognition, (b) information gathering, (c) involving others in delivering care, and (d) delivering patient-focused care. We discuss these categories in turn below.

### Category 1: Uncertainty Around Symptom Recognition

This first category depicts the process participants underwent in trying to work out the symptoms described in the case vignettes, particularly the hypoactive case details, as this case was less specific in its presentation than the hyperactive case. The discussion was taken up with the participants trying to interpret the symptoms described, and suggestions were offered as to what the symptoms manifested. One suggestion was the symptoms might be interpreted solely in terms of “normal” poststroke experience, as seen in the extract below:

Is it possible that some of the fatigue and apathy may also be a “normal” grieving response to the loss of function resulting from the stroke?

Rather, however, than viewing symptoms as a usual part of the poststroke journey, most participants offered other suggestions. The possibility of cognitive impairment was commonly raised as a possible explanation of the symptoms, with participants referring to the possibility of premorbid dementia, particularly when discussing the hypoactive case:

I would think she has mild dementia and has been taken out of her familiar setting of home and this has knocked her off a bit.

I would consider whether these are signs of early stages of dementia.

This lady certainly appears to have cognitive decline and her drowsiness may be part of a dementia picture.

More commonly, however, than referring to possible dementia, participants tended to use the somewhat more generic term “confusion,” as in the following instances:

We have patients who have language, processing or visual difficulties due to the stroke however they are described as “confused.”

I don’t particularly like the word confused as I do find people are labelled confused and can be sometimes written in medical notes as “?dementia” . . . Members of the MDT, family and patients often do not understand the different aspects of cognition but just identify the patient is confused.

As is evident above, even in using the description “confused,” participants expressed some dissatisfaction with the use of this term. One reason provided, as above, was that the term failed to distinguish between different elements of cognition. It was thus regarded often as a description that failed appropriately to identify the symptoms seen in the potential patient, as seen below:

I think confused is too broad a term and doesn’t identify the reasons for the behaviors.

[Confusion] means very little, staff need to expand what why and how it affects them, it is often used as a word with little meaning attached.

As noted above, two participants did refer to delirium in the course of the discussions:

We certainly are doing a lot of work at the moment with medical and nursing front line staff to recognize that it is a medical emergency . . . we have worked very hard here to “Think delirium.”

We had a very useful in-service on Delirium recently outlining what to look out for as compared to a longer-standing cognitive impairment. We were told to look out for delirium as an acute onset and fluctuating course with inattention and either disordered thinking or an altered level of consciousness.

What, however, is especially interesting in these two cases is that the participants described only their own experiences of receiving training in relation to delirium: They did not in the course of the discussions explicitly offer delirium as an explanation of the symptoms described in the vignettes. This suggests some hesitation or uncertainty on the part of these participants in applying this diagnosis in the current instances. Although, therefore, these extracts indicate potentially greater awareness of the possibility, there remains an element of uncertainty as to identification of delirium in specific cases.

What is seen then in these extracts is that in discussing the symptoms described, only a minority of participants made any reference at all to the possibility of delirium. Even in these instances, however, the possibility of delirium was not pursued. For the most part, participants instead offered more generalized descriptions in terms of cognitive impairment, dementia, or commonly confusion. Although many participants remarked on the difficulties associated with the term “confusion,” it nonetheless provided a recurring suggestion for how to understand the symptoms being discussed.

### Category 2: Information Gathering

In the absence of any confidence in arriving at an explanation of symptoms, as seen above, the need for further information emerged as a recurring aspect of the discussions. Participants suggested a range of cognitive screening tools such as the Mini Mental State Examination, the Addenbrook Cognitive Examination (ACE-III), or the Montreal Cognitive Assessment (MoCA) that might be deployed to gather further information. Some participants suggested carrying out assessments that were unique to their specific professions, such as functional assessments or comprehension/language assessments. More usually, participants referred to an action or several actions to be taken in response to the symptoms presented as a means of establishing a clear clinical picture. These actions created a direct link with Category 3 on involving other clinicians in the management of care, as professionals referred to and discussed matters with their colleagues. The actions proposed are seen in the context of the participants’ own professional disciplines: The different professional roles emanate in the description of the actions to be taken. Thus, as seen in the extract below, the participant, a nurse, refers to assessment of any physiological changes and ensuring basic care needs are met (hydration, medication, bladder, and bowel function):

I would measure her observations including temperature I would also dip stick her urine looking for infection, check urea and electrolytes and Liver function. I would be keeping a close eye on her throughout the day and night . . . I would also do bloods looking for possible infection and dehydration. I would also keep a check on bowel movements.

Participants were particularly interested in working out possible causes for the “confusion”: changes in the type or timing of medication or whether there was an underlying infection which triggered a discussion around asking doctors or nurses to check inflammatory markers, particularly for a urinary tract infection. Once the discussion turned to examine the possibility of an infection, five participants wondered whether the symptoms manifested in the vignette were describing a delirium. This line of discussion, however, did not go beyond the context of exploring the possibility of an underlying infection and, similarly to the extracts seen in Category 1, used centered on the possibility of “confusion” despite difficulties in the use of the term rather than offering up other possibilities.

The two participants who had introduced the possibility of delirium into the discussion here suggested the use of the specific delirium rapid assessment tool The 4AT (Healthcare Improvement Scotland, 2014). The first came in relation to the hypoactive delirium case and was the only suggestion related to delirium screening for that case:

On admission the 4AT screening for dementia/delirium should have been done, so you could track the changes.

Another participant suggested that the 4AT tool be used in relation to the hyperactive delirium case:

We use the 4AT screening tool for delirium and cognitive impairment. This should be carried out on all patients on admission . . . I think you need to be careful using this measure particularly if you do not have a clear idea of whether there was a degree of cognitive impairment prior to admission.

As seen in the extract above, however, the participant did not express full confidence in this tool for the identification of delirium. The participant argued that it needed to be used with care and that, without appropriate information as to the previous state of the patient, the outcomes of the test might be of uncertain value in any investigation of symptoms.

Thus, for all participants, the need for further information was a key part of attempting to arrive at a more precise explanation of the symptoms as described. Participants’ searches for information closely mirrored their initial responses to the symptoms as presented: Initial suggestions of delirium were linked to tools that might confirm such suggestions, while more generalized descriptions of symptoms could lead to broader and less-directed searching for further information on the patients.

### Category 3: Involving Others in Delivering Care

All participants considered ongoing communication with other team members as well as with the family or caregivers as key to managing the care of the patients in the vignettes. One element of this comprised the roles of the different professionals in clinical settings. Whereas participants described themselves as working collaboratively with other clinicians and with family members in providing care for the patients, they did not regard all members of the team as equally responsible for all that this involved. In particular, participants saw themselves and other members of the team as having different levels of involvement with identification and diagnosis of the patient’s symptoms and the provision of subsequent care. Thus, as seen below, one participant argued that, notwithstanding that early identification of symptoms was a shared responsibility, in practice, this responsibility fell to nursing staff:

I think it’s everyone’s responsibility but realistically the screening is done by nursing staff . . . as part of the admission process and it’s then ongoing.

The primary involvement of nurses in screening was regarded as deriving largely from their having greater involvement than other team members with patients in the early stages of poststroke recovery, placing them in a better position than others to notice and respond to difficulties that a patient might be experiencing:

If a patient is unwell or appears confused, OT /PT and SLT will defer therapy until the patient is able to participate so in most cases, the chances are it would be nursing staff . . . who would notice any confusion/delirium.

Thus, participants did not view other clinicians as having the same level of familiarity with patients’ difficulties at that stage. The role of other team members, then, was one of feeding back any observations to the team as a whole and ensuring that these observations were properly recorded:

As a physiotherapist I am not routinely carrying out formal screening for delirium we do note changes in behavior and ability to participate in treatment and feed this back promptly to the MDT and document in the patient notes.

Conversely, the participants described roles that were very much directed toward involving members of the patient’s family in the poststroke journey. This could comprise both efforts to get family members to provide the patient with a break from the clinical environment or to involve them in the actual provision of treatment itself:

Could she be encouraged to join in any activities with other patients or perhaps her family could take her out for a short trip.

In my unit the MDT readily involve family members to assist in our treatment sessions and this can work really well.

Here, family were regarded not just as offering some distraction for the patient or assistance in the delivery of treatment, but were described as a key part of the process of rehabilitation:

Encourage someone in, use stimulation as needed, use family to help engage.

It may be good to involve her family in therapy to see if they can get her to engage.

I wonder if a family contact and a joint session with family may help with engagement. In the rehab ward this has often be a useful tool as patients sometimes find it more engaging and stimulating with someone familiar there.

As seen in the extracts above, the central concern here for participants in all roles within the team was one of facilitating the “engagement” of the patient with what was going on around him or her. Thus, although all participants described their roles as contributing to different extents to the early identification of symptoms and diagnosis, they at the same time referred to the importance of team-working in efforts to deliver therapy and promote rehabilitation and in seeking to involve members of the patient’s families in these broader steps toward encouraging recovery.

### Category 4: Delivering Patient-Focused Care

The final category is one that recurred throughout the discussions examined here: the importance that participants attached to providing patient-focused care. There was a clear picture of all practitioners’ values and regard for a patient’s well-being. One part of this was directed toward practical steps that would both aid the patient in his or her stay in the clinical setting and also facilitate the engagement of the patient in being an active participant in that environment. In the following extracts, the participants refer to the importance of ensuring that the patient had available items that were necessary on a day-to-day basis:

Ensuring the patient has their hearing aid, glasses, mobility aid etc. is crucial.

Ensure that mobility is gently encouraged and that adequate pain relief is available to allow this. Orientating the patient with a clock and familiar items may also help. Hearing aids and glasses should be in use if required.

Providing care on a practical basis also required attention to the physical surroundings, including placing the patient in a location that might facilitate greater participation in the setting and thereby lead to increased engagement with what was going on:

I would like to see if she could be encouraged to eat and drink more if she was taken to a table to eat with others. Eating on your own by your bedside does not encourage an appetite or socialization.

Of course, delivering patient-focused care was seen as involving more than simply the organization of practical arrangements. Appropriate care involved at least as much attention to the emotional concerns and needs of the patient to encourage psychological rehabilitation. Part of this involved recognizing and responding to the concerns and fears of the patient who has found himself or herself in a clinical environment and is potentially unaware of these surroundings and what might lie ahead:

Is she aware of where she is and what has happened or is she perhaps just scared . . . with her declining memory does she know what has happened to her and what the future may hold. Even if she has been told she may not remember or feel part of the process.

Important to discuss with patient any reason why she is feeling like this. What are her goals during her hospital admission? Perhaps she thinks she may not be able to return home and maybe depressed at being in hospital.

What the participants regarded as important in responding to concerns raised by or potentially held by the patient was to help him or her to understand what had happened and to provide the appropriate reassurance:

I would ask her if there is anything worrying her, making her feel so tired and reassure her and encourage other patients if possible to interact with Mrs. B. Explaining what has happened and outlining what can happen and what can be done, may help Mrs. B understand better, what is going on.

And, offering reassurance was viewed as key to alleviating the concerns of the patient and ensuring that the care provided did take the patient to be the central focus of that care:

I would . . . offer reassurance and try make him feel safe and cared for in his surroundings.

Thus, for all participants, a focus on the patient as the person receiving care that was oriented to practical aspects of their stay in the clinical environment and that addressed psychological concerns and broader issues of well-being was of primary importance in helping the patient along the poststroke journey.

## Discussion

We set out to explore the ways in which clinicians working in interprofessional stroke-unit teams understood and would respond to potential delirium in stroke survivors. The present study was conducted with a small sample of participants and in a specific location, namely, Scotland, UK. Nonetheless, the present findings can inform efforts, both there and more widely, to facilitate the early identification of delirium in stroke survivors by clinicians in interprofessional teams.

In this study, clinicians’ understandings of delirium varied, particularly in response to the description of symptoms associated with a hypoactive delirium. Lack of knowledge of delirium symptoms and lack of confidence in its identification are consistently reported in nursing and medical literature in the United Kingdom and beyond (Davis & MacLullich, 2009; Rice et al., 2011; Ryan et al., 2013). This is heightened in cases of hypoactive delirium (Bellelli et al., 2014; McCrow et al., 2014). An important consideration, which conveys the clarity of knowledge and understanding of delirium, is around the use of language to discuss delirium; as seen in our sample, most of the clinicians tended to use tentative language to describe delirium symptoms, using the terms “confusion” and “delirium” interchangeably. This is confirmed in other acute hospital settings: A review of case notes by Day et al. (2008) confirmed a near absence of the word “delirium” within patient notes. Rice et al. (2014) confirmed these findings as they reported that nurses equated the symptoms of delirium with “confusion” documenting this in patient’s notes, rather than any observable delirium features.

A minority of participants in this study stated explicitly that they had received training to “Think Delirium,” as per the Healthcare Improvement Scotland (2014) initiative to help clinicians improve identification and initial management of the condition. And, as seen above, these two participants referred to delirium in the discussions of the cases described in the vignettes. Noticeably, however, even following this training, the participants did not apply this term to the case being described or seek to suggest the possibility to other clinicians involved in the discussions. Further work therefore might usefully explore the impact this initiative has had on clinicians’ ability to recognize a delirium in stroke survivors, given the known challenges of identification of the condition in this area of practice (Infante et al., 2017; Lees et al., 2013). While the present study was not concerned with delirium education *per se*, elements of this emerged from our findings. In a systematic review of 26 studies, Yanamadala et al. (2013) found that interactive, multistrategy teaching programs resulted in improved staff recognition of delirium, but in this review, the emphasis was not on interprofessional education, as only a few of the studies included in the review had more than one discipline in the participant mix. There are multiple reasons that may explain the long-term success or lack of success of delirium educational programs, but in a field where it is clear that interprofessional working is important in achieving good outcomes for patients (Godfrey et al., 2013), it stands to reason that collaborative practice should come as a result of professionally inclusive approaches to delirium education, which could potentially result in improvements to patient outcomes (Sockalingam et al., 2014). Given that early identification of delirium requires that clinicians have knowledge of delirium symptoms and the confidence to identify delirium in clinical practice, there remains a need for training that specifically equips clinicians with the confidence to apply their knowledge and to share it with other members of the clinical team.

It is important to note that despite the inconsistent use of the term *delirium*, the present findings indicate that the key principles of initial delirium management would be followed even if accurate recognition was not achieved: Participants presented some of the key features of patient-centered care throughout the discussions, referring to a caring, compassionate, and holistic approach. Participants also described their practice in attempting to identify a physiological cause, reorientation, and engaging with family and caregivers, actions that are all consistent with U.K.-wide best practice guidelines (Healthcare Improvement Scotland, 2014; National Institute for Health and Care Excellence, 2010, 2014). Family or caregivers were regarded as an important part of the team, not only as potential informants but also as contributors to the therapeutic process. The role of family is recognized as part of the health care team interventions in the management of delirium, whether it is as informants of preadmission cognitive function (Healthcare Improvement Scotland, 2014) or as part of multicomponent interventions as family and caregivers could play a role in reorientation, cognitive stimulation, and sensory function (Martinez et al., 2012). All such potential contributions point to the importance for the clinical team of involving family members and/or caregivers in the therapeutic process from an early stage, potentially all the more so in circumstances that are otherwise marked by uncertainty and in which the gathering of available information about the stroke survivor is a priority. Although professional boundaries often inhibit meaningful involvement of family members in the delivery of care (Omori et al., 2019), the informal but practical knowledge that family members bring can usefully inform the care that will be of most immediate benefit to a loved one (James et al., 2009). At the same time, involvement of family members from an early stage will allow them to feel less “in the dark” about what has happened to a loved one who has become less familiar and potentially absent from them (Day & Higgins, 2015, p. 1712).

In terms of interprofessional working in the stroke unit, the divisions of roles within teams clearly arose as well as a sense of distinction in the perceived knowledge base between each professional discipline. The division of roles among some members of interprofessional teams emerged when discussing roles around delirium identification. While it is widely recognized that nurses are key players in delirium recognition (Dahlke & Phinney, 2008; Fick et al., 2007, 2013; Hall et al., 2012), the roles of other professionals is, to the best of our knowledge, not described in the literature on delirium recognition. Single studies on delirium education which included other professional disciplines such as occupational therapy and physiotherapy within their cohorts did not report on the specific competencies or roles of different professional groups in delirium identification and management (Bellelli et al., 2014; Foster et al., 2010; Godfrey et al., 2013; McAiney et al., 2012; Teodorczuk et al., 2013). In a Scottish context, occupational therapists are regarded as experts in cognitive assessment and their role in this area of stroke care is recognized in best practice guidelines (Scottish Intercollegiate Guidelines Network, 2010). More broadly, however, particular members of clinical teams might well be equipped to contribute to assessment in a range of contexts. Indeed, one of the nurse participants in this study reflected upon perceived skills and role in cognitive assessment, yet in discussing cognitive screening, the occupational therapists in this study did not mention delirium screening specifically, nor did they consistently use accurate language to discuss the symptoms of a delirium in a stroke survivor. Two participants in our study discussed the use of a rapid delirium assessment tool, the 4AT. This tool is designed to be used in routine care by any professional as a means of triggering comprehensive diagnostic processes (Healthcare Improvement Scotland, 2014). Screening for the condition is an important first step in arriving at a delirium diagnosis and this can be done by any team member using a suitable tool such as the 4AT. Clarke and Forster (2015) observed that interdisciplinary team work goes beyond different professionals working together but rather it implies an accepting of responsibility of the group effort on behalf of their patients. Indeed, one of the nurse participants felt that it is everyone’s responsibility to be able to recognize the symptoms of delirium, a matter which is discussed in contemporary literature on delirium as there is a clear argument that delirium recognition should be a shared concern for all members of the team (Bellelli et al., 2014; Godfrey et al., 2013).

There remains the question of whether accurate recognition and clinicians’ use of language are indeed crucial in the management of delirium. Teodorczuk et al. (2012) argued that the lay term “confusion” is unhelpful as it is used interchangeably as both a symptom and a diagnosis. Others warned that the use of the term “confusion” is misleading and may lead to either misdiagnosis or mismanagement of delirium (Fleet et al., 2015; Morandi et al., 2012; Teodorczuk et al., 2013). As Sheng et al. (2006) point out, early identification of delirium is all the more important for stroke survivors: “early awareness of the incidence of delirium in stroke patients may lead to better management of delirium patients, particularly elderly patients, which may in turn improve the prognosis of those patients” (p. 1197). The issue then is not that imprecise use of language will necessarily result in delivery of care that is inappropriate in clinical settings: The present participants’ references to seeking further information, involving other team members in the management of care, and putting the patient at the center all suggest otherwise. Rather, the concern is that all such steps will result in delay in providing the optimal care that is required to facilitate the best outcomes for stroke survivors. And, as seen in the findings from the present study, addressing this delay remains a challenge.

This is the first study to reveal the ways in which professionals of different disciplines working in acute stroke units understand and might respond to delirium in stroke survivors. Participants included members of a range of professions working in these settings. One limitation of this study is that despite various attempts to recruit doctors to the study, none came forward to participate. Further work is needed to examine how medical staff make sense of their own roles and those of other professionals working in interprofessional teams to identify cases of delirium in stroke survivors. The composition of the research team, however, ensured that a range of individuals from different backgrounds were involved in and agreed the analysis and findings of the study, with team members coming from the fields of occupational therapy, applied human physiology, neurological rehabilitation, geriatric medicine, and health psychology. The present findings thus usefully demonstrate the understandings of a broad range of professionals involved in the day-to-day interprofessional care of patients with delirium following stroke.

## Supplemental Material

supplemental_file_Staff_response_to_delirium_in_acute_stroke_25082020 – Supplemental material for Identifying and Responding to Delirium in Acute Stroke: Clinical Team Members’ Understandings Click here for additional data file. Supplemental material, supplemental_file_Staff_response_to_delirium_in_acute_stroke_25082020 for Identifying and Responding to Delirium in Acute Stroke: Clinical Team Members’ Understandings by Gail Carin-Levy, Kath Nicol, Frederike van Wijck, Gillian Mead and Chris McVittie in Qualitative Health Research
