# Supplementary material for: Identifying and Responding to Delirium in Acute Stroke: Clinical Team Members’ Understandings
Source: Qual Health Res. 2020 Sep 24;31(1):137–47. doi: 10.1177/1049732320959295 (PMC7750676; doi:10.1177/1049732320959295)
Supplement: supplemental_file_Staff_response_to_delirium_in_acute_stroke_25082020 – Supplemental material for Identifying and Responding to Delirium in Acute Stroke: Clinical Team Members’ Understandings [file supplemental_file_Staff_response_to_delirium_in_acute_stroke_25082020.pdf]

Identifying and responding to delirium in acute stroke: clinical team members' understandings – Vignettes

Trigger Question	Vignette 1	Vignette 2
**What are your initial thoughts?**	Mrs. B is an 82 year old lady who was admitted recently with acute weakness of the left side of her face and her left arm. She was diagnosed clinically as having had a right sided infarct. Computed tomography (CT) of her brain confirmed an R hemisphere lacunar ischaemic stroke, as well as mild small vessel disease and generalized atrophy reported as consistent with her age.<br>On the third night of her admission she became confused and restless, but this has resolved by morning.	Mr. A is an 82 year old man who was admitted recently with acute weakness of the left side of his face and his left arm. He was diagnosed clinically as having had a right sided infarct. Computed tomography (CT) of his brain confirmed an R hemisphere lacunar ischaemic stroke, as well as mild small vessel disease and generalized atrophy reported as consistent with his age. On the third night of his admission he became confused and restless, but this has resolved by morning.
**How do you interpret this?**<br><br>**What does this information add?**	Discussion with family members reveals that they have become a little worried about her memory recently. In the last 6 months, Mrs. B has forgotten to pay a few bills, and the family had to help her with this. On the recommendation of the GP, the family have also arranged for her medication to be dispensed in blister packs via the local pharmacy	Discussion with family members reveals that they have become a little worried about his memory recently. In the last 6 months, Mr. A has forgotten to pay a few bills, and the family had to help him with this. On the recommendation of the GP, the family have also arranged for his medication to be dispensed in blister packs via the local pharmacy.
**What do you make of this?**	The night time confusion seems to have resolved, but now, Mrs. B is rather drowsy by day and spends much of the time asleep. She doesn't seem to want to engage with physiotherapy during the day and appears apathetic.	The night time confusion continues, but now, Mr. A is also confused during the day. He recently shouted at one of the physios who tried to take him to the gym. Family members reported this is completely out of character.
**This is all the information currently available to you. What's your view about the patient's mental state?**	Due to Mrs. B's drowsiness, the nurses are concerned that she is not taking adequate fluids and IV fluids were commenced. It appears from the notes that Mrs. B has not had any bowel movements since admission.	Mr. A has not been taking adequate fluids and has commenced IV hydration. This is made complicated by his repeatedly pulling out the drip. One of nurses reported that Mr. A appeared at times to be responding to unseen stimuli.
